# *Plasmodium falciparum* parasites overexpressing farnesyl diphosphate synthase/geranylgeranyl diphosphate synthase are more resistant to risedronate

**DOI:** 10.1590/0074-02760180174

**Published:** 2018-08-13

**Authors:** Heloisa B Gabriel, Mauro F Azevedo, Emília A Kimura, Alejandro M Katzin

**Affiliations:** 1 Universidade de São Paulo, Instituto de Ciências Biomédicas, Departamento de Parasitologia, São Paulo, SP, Brasil; 2 Universidade Federal de São Paulo, Departamento de Biociências, Santos, SP, Brasil

**Keywords:** P. falciparum, FPPS/GGPPS, risedronate, overexpression

## Abstract

Farnesyl diphosphate synthase/geranylgeranyl diphosphate synthase (FPPS/GGPPS) is a key enzyme in the synthesis of isoprenic chains. Risedronate, a bisphosphonate containing nitrogen (N-BP), is a potent inhibitor of blood stage *Plasmodium*. Here, we show that *P. falciparum* parasites overexpressing FPPS/GGPPS are more resistant to risedronate, suggesting that this enzyme is an important target, and bisphosphonate analogues can be used as potential antimalarial drugs.

It has been estimated that more than 200 million cases of malaria occur annually, resulting in over 400,000 deaths.^1^ Among the five *Plasmodium* species that infect humans, *P. falciparum* results in most cases of morbidity and mortality. Chemotherapy is an important component of control strategies, and the looming resistance against artemisinin and its derivatives, which are the most effective antimalarial drugs, is a serious challenge to the goal of the World Health Organization for the reduction of malaria cases and deaths.^1^ Isoprenoid synthesis is a metabolic pathway essential for parasite survival during the erythrocytic cycle and is therefore a potential target for the development of antimalarial drugs.^2^ ^-^ ^3^ An essential step in the synthesis of all isoprenoids is the elongation of the isoprene chain by prenyltransferases, which are classified according to the chain length of the final product and the stereochemistry of the double bond formed by condensations. Among the prenyltransferases, farnesyl diphosphate synthase (FPPS) and geranylgeranyl diphosphate synthase (GGPPS) are the most studied isoprenoid-modifying enzymes in *Plasmodium*.^4^ ^-^ ^5^

The biosynthesis of farnesyl diphosphate (FPP) and geranylgeranyl diphosphate (GGPP) is catalysed by a single bifunctional enzyme (FPPS/GGPPS) in *P. falciparum* and *Toxoplasma gondii*.^4^ ^-^ ^6^ The metabolites generated are the main precursors of all secondary products from isoprenoid pathways such as those for vitamin E,^7^ vitamin K,^8^ carotenoids,^9^ ubiquinones,^10^ and dolichols.^11^

Bisphosphonates are inhibitors of bone resorption applied for the treatment and prevention of osteoporosis.^12^ Risedronate, a bisphosphonate containing nitrogen (N-BP), inhibits *P. falciparum* FPPS/GGPPS enzymatic activity *in vitro* and has potent activity against blood stages when added to parasites during *in vitro* cell culture.^4^ The inhibitory effect induced by risedronate can be partially reversed by the simultaneous addition of FPP or GGPP during *P. falciparum* culture treatment.^13^ A similar inhibitory effect was reported *in vivo* against murine malaria parasites. In the same study, it was also demonstrated that risedronate was not toxic for animals.^13^ In addition, a synergistic effect with nerolidol, a terpene approved by the Food and Drug Administration (FDA) as a food-flavouring agent, demonstrated the potential use of risedronate in combination therapies.^14^ ^-^ ^15^ It has recently been demonstrated that FPPS/GGPPS presents different binding sites for promising new drugs against malaria.^16^ In this study, we engineered *P. falciparum* parasites that overexpress FPPS/GGPPS and evaluated its sensitivity to risedronate to investigate whether FPPS/GGPPS is its main target in *P. falciparum*.

Since FPPS/GGPPS is constitutively expressed in *P. falciparum*,^4^ initial attempts aimed to overexpress FPPS/GGPPS-green fluorescent protein-haemagglutinin (FPPS/GGPPS-GFP-HA) under the control of the EF1-a promoter. Stably transfected parasites were never recovered, suggesting that overexpression of FPPS/GGPPS-GFP-HA might be toxic. Previous studies have demonstrated toxic effects due to the overexpression of enzymes that use GPP or FPP as substrates,^17^ ^-^ ^18^ caused mainly by the depletion of FPP substrate. In order to keep the overexpression to minimum during the selection of transfected parasites, FPPS/GGPPS was cloned into pRM2-GFP-HA-DD24 (Fig. 1A).^19^ The expression as a fusion with HA and the destabilisation domain (DD) would target the fusion protein for degradation, which could only be prevented by the presence of the ligand Shld-1.^20^ This strategy was successful, and stable transfection in 3D7 strain parasites generated the transgenic line FPPS/GGPPS-DD-epi.

Given that the transgene in pRM2-derived vectors is under the control of the MSP2 promoter, which is strongly active in schizonts, both RNA and proteins were extracted from transgenic and wild-type (WT) parasites at this stage to compare their expressions. FPPS/GGPPS transcript levels were compared by real-time quantitative-polymerase chain reaction (RTq-PCR) (Fig. 1B). As expected, the extra copies of FPPS/GGPPS gene in the transgenic line resulted in significant overexpression, with transcript levels being about 2-fold higher than 3D7 parasites (Fig. 1B). To verify that DD/Shld-1 regulation played a role, protein was extracted from parasites cultured with (400 nM) or without Shld-1 and analysed by western blotting using antibodies against the HA epitope (Sigma-Aldrich, St. Louis, MO, USA), or PTEX150,^21^ which was used as an internal control. FPPS/GGPPS-HA-DD24 was only detected in the sample extracted from parasites maintained on Shld-1, demonstrating that the ligand was required to stabilise the protein (Fig. 1C). Due to the lack of a specific antibody, it was not possible to compare FPPS/GGPPS expression between transgenic and WT parasites at the protein level. However, considering the increased transcript levels and that FPPS/GGPPS-GFP-HA can be easily detected from parasites cultured on Shld-1, this enzyme was likely expressed at higher levels in the transgenic parasites cultured on Shld-1 than in WT parasites.

To investigate whether FPPS/GGPPS expression correlates with resistance to risedronate, 3D7 WT and transgenic parasites at the ring stage were cultured with different concentrations of the drug for 48 h to determine the IC_50_ (Fig. 2). Growth was determined by fluorescence method^22^ and confirmed by microscopic examination.^23^ Shld-1 (400 nM) was added after 24 h to stabilise the FPPS/GGPPS-HA-DD24 fusion since it was supposed to be expressed in schizonts. The IC_50_ of 3D7 and transgenic parasites cultured without Shld-1 was about 20 µM, suggesting that the transfection process and expression of the selectable marker hDHFR did not affect sensitivity to risedronate. However, in the presence of Shld-1, the IC_50_ of transgenic parasites increased 1.7-fold to about 34 µM, while the sensitivity to risedronate of 3D7 parasites was not affected. Sensitivity to the unrelated drug chloroquine was neither affected by the ligand nor by FPPS/GGPPS overexpression (Supplementary data). Lower Shld-1 concentrations did not affect the IC_50_, and higher concentrations were not evaluated since they were toxic to the parasites, thereby reducing their growth by about 11% per reinvasion cycle.^19^

Although FPPS/GGPPS-DD-epi parasites have an IC_50_ significantly higher than that of WT parasites, the resistance phenotype was subtle when compared to the phenotype of other transgenic lines. Overexpression of octaprenyl pyrophosphate synthase/phytoene synthase (OPP/PSY) caused a 5-fold increase in the IC_50_ to squalestatin.^24^ It was possible that the achieved FPPS/GGPPS overexpression was not as strong as the OPP/PSY overexpression, which could be due to incomplete protein stabilisation in the presence of Shld-1 or lower plasmid copy number.

The results presented here corroborates previous findings that FPPS/GGPPS is an important target of risedronate in *P. falciparum*, suggesting that this compound or a more potent analogue could be developed as an antimalarial drug or be applied in combination therapies in future.

For the plasmid construction, the FPPS/GGPPS coding sequence (PlasmoDB ID: PF3D7_1128400) was synthesised by GenScript, and FPPS/GGPPS was cloned in pRM2-GFP-HA-DD24,^19^ replacing the GFP gene with the XhoI/MluI restriction site to generate pRM2-FPPS/GGPPS-HA-DD24. Cultures of *P. falciparum* clone 3D7 were grown as described previously,^25^ except that human serum was replaced with Albumax I (0.5%; Invitrogen/Life Technologies, Carlsbad, CA, USA). Parasite multiplication was monitored by microscopic evaluation of Giemsa-stained thin smears. Schizont stages were purified with magnetic columns [magnetically activated cell sorting (MACS) separation columns; CS; Miltenyi Biotec].^26^ Parasites were transfected as previously described^27^ using the electroporation conditions established elsewhere.^28^

**Fig. 1:**
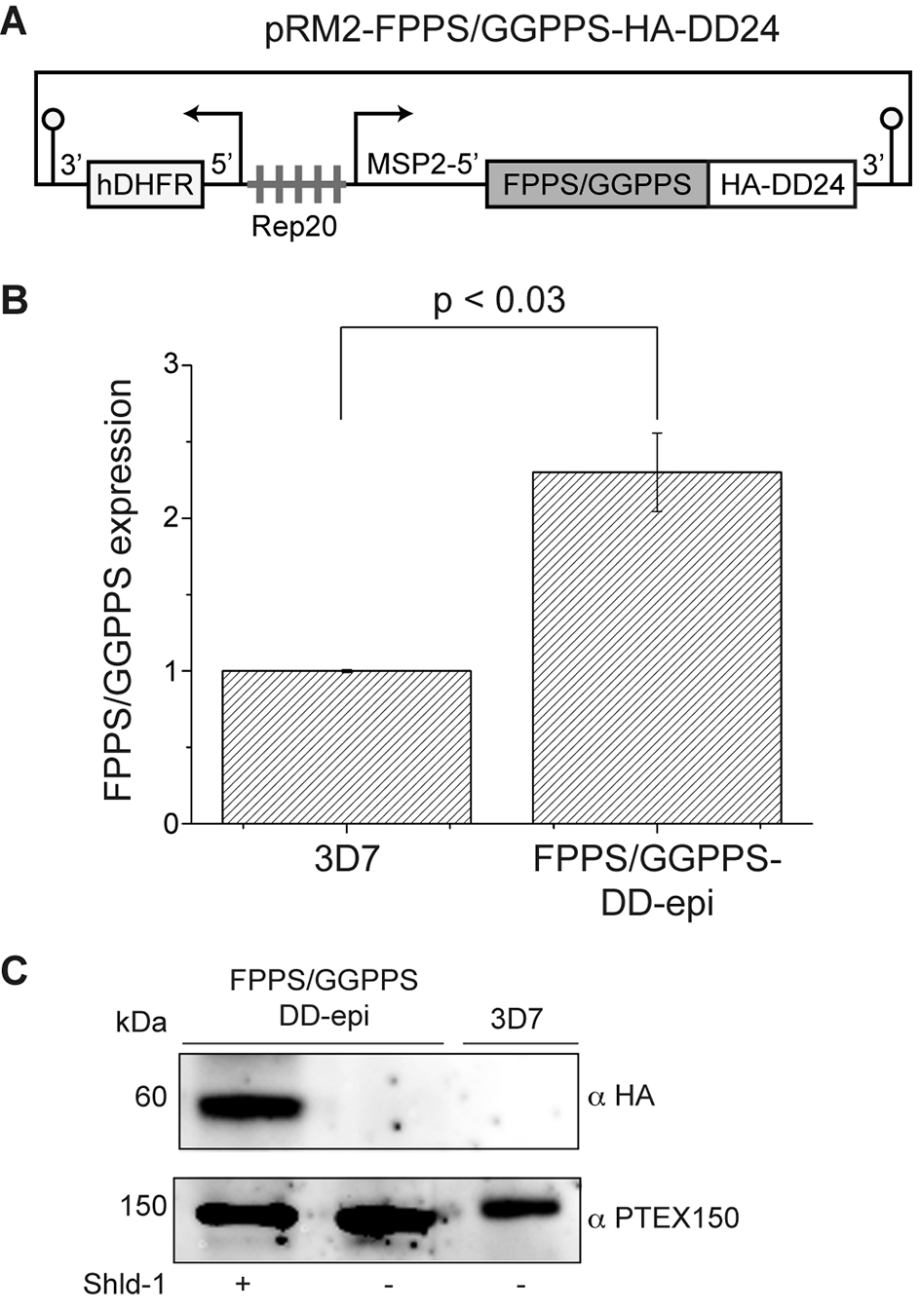
overexpression of farnesyl diphosphate synthase/geranylgeranyl diphosphate synthase-destabilisation domain-epi (FPPS/GGPPS-DD-epi). (A) Schema of plasmid construction used for transfection. (B) Real-time-polymerase chain reaction (RT-PCR) showed overexpression of FPPS/GGPPS-DD-epi compared with wild-type 3D7. (C) Western blot analysis showing the expression of FPPS/GGPPS protein only when Shld-1 was added. Antibody for a constitutively expressed protein (PTEX150) was used as an internal control. Band intensities were measured by ImageJ, density values were evaluated by one-way analysis of variance (ANOVA), and p values are indicated.

For the cDNA preparation, the RNA was extracted using TRIzol LS (Invitrogen) following the manufacturer’s instructions. Oligonucleotides (GAGTGGGAAAAAGTGGCTTG and CACATCATTCACCGCATTCT) for the detection of FPPS/GGPPS were designed using Primer3 (http://frodo.wi.mit.edu/). The internal control transcript used for calibration throughout the experiments was locus seryl t-RNA transferase (PlasmoDB no. PF3D7_0717700), previously shown as a reliable control.^29^ The relative mRNA expression was obtained using the formula 2^*−ΔCT*^ . All experiments were performed in duplicate.

**Fig. 2:**
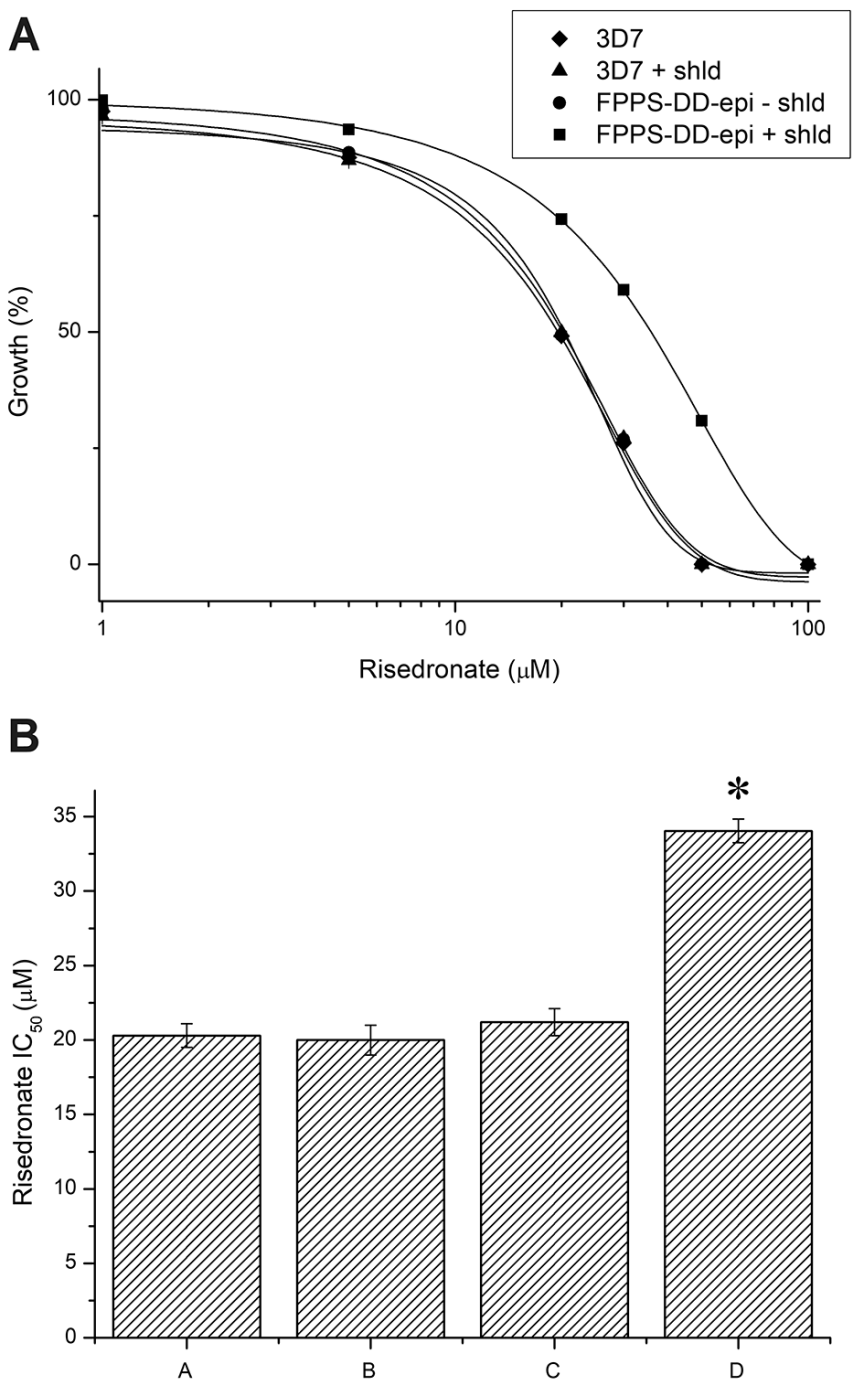
parasites overexpressing farnesyl diphosphate synthase/geranylgeranyl diphosphate synthase (FPPS/GGPPS) were more resistant to risedronate. Parasites of the wild-type 3D7 strain and the transgenic line FPPS/GGPPS-destabilisation domain-epi (FPPS/GGPPS-DD-epi) cultured for two days in the presence of various concentrations of risedronate. (A) Nonlinear regression of parasite growth and risedronate concentrations. (B) The IC_50_ represents the means with 95% confidence intervals from three experiments: A-3D7 without Shld-1 20.3 ± 0.8 µM, B-3D7 with Shld-1 20 ± 1 µM, C-FPPS/GGPPS-DD-epi without Shld-1 21.2 ± 0.9 µM, and D-FPPS/GGPPS-DD-epi 34.8 ± 0.8 µM. Statistical significance was determined by one-way analysis of variance (ANOVA), *p < 0.02.

In the inhibition tests, risedronate was dissolved in water, resulting in 25 mM stock solutions.^13^ WT and transgenic parasites at the ring stage were cultured in different concentrations of the drug (200, 20, 2, 0.2, and 0.02 µM) for 48 h. Growth was determined by the SYBR Green method^22^ and confirmed by microscopic examination.^23^. Shld-1 (400 nM) was added after 24 h to stabilise the FPPS/GGPPS-HA-DD24 fusion. All tests were performed in triplicates from three independent experiments. The IC_50_ for growth inhibition was calculated by nonlinear regression in GraphPad Prism 5.0 (GraphPad Software, Inc., San Diego, CA, USA).

For western blot analyses, synchronous cultures of 3D7 and transfected parasites with and without Shld-1 at schizont stages were treated with 0.15% saponin in RPMI media and washed twice with PBS. Proteins were extracted from the parasite pellets after resuspending in buffer containing 0.05 M Tris-HCl (pH 6.8), 10% glycerol, 2 mM EDTA, 2% SDS, 0.05% bromophenol blue, and 50 mM dithiothreitol^30^ for separation by sodium dodecyl sulphate-polyacrylamide gel electrophoresis (SDS-PAGE). The α-HA monoclonal antibody (1:500 dilution; Sigma-Aldrich) was used, and α-PTEX150 (1:1000)^21^ was used as a control.
